# Erratum: Electrophysiological markers of fairness and selfishness revealed by a combination of dictator and ultimatum games

**DOI:** 10.3389/fnsys.2023.1186493

**Published:** 2023-03-27

**Authors:** Frontiers Production Office

**Affiliations:** Frontiers Media SA, Lausanne, Switzerland

**Keywords:** cooperation, EEG, N1, P2, MFN, late positive potential, spiteful punishment, costly punishment

An omission to the funding section of the original article was made in error. The following sentence has been added: “Open access funding was provided by the University of Lausanne.”

The publisher apologizes for this error. The original article has been updated.

